# The Foundations of Virology: Discoverers and Discoveries, Inventors and Inventions, Developers and Technologies

**DOI:** 10.3201/eid1904.130054

**Published:** 2013-04

**Authors:** Sharon Bloom

**Affiliations:** Author affiliation: Centers for Disease Control and Prevention, Atlanta, Georgia, USA

**Keywords:** virology, viruses, history of medicine, foundations of virology, discoverers and discoveries, inventors and inventions, developers and technologies

History of science is not a priority of most virologists. However, if given a visually engaging coffee-table book of the persons and discoveries that shaped the field of virology since Hippocrates, curious scientists will inevitably start paging through history. For this reason, every chairperson of virology departments worldwide should leave a copy of Fred Murphy’s The Foundations of Virology in the break room.

Professor Murphy has a long and distinguished career in emerging viruses from the perspective of public health and academic bench science. Murphy has made his 536-page book available as a downloadable, lower-resolution, eBook on his website, which for years has also been an enormous open-access library resource for virologists. Because of numerous requests to have hard copies printed for fingertip access, Murphy has made this book available in paperback. There are clear advantages to having the hard copy in hand, in a place where it can be picked up and absorbed a little at a time.

The full title of the book reflects the content; it is a chronology of images of discoverers, developers, and inventors, alongside their discoveries, developments, and inventions. The book is physically laid out in a landscape format, usually with multiple photographs on each page, featuring scientists, their institutions, spot maps from major epidemics, electron micrographs of then-emerging viruses, and graphics from landmark publications. Along the bottom of each page is printed in large font the year, names of contributors, and their historic contributions. This bottom line layout serves as an effective design enabling quick orientation to time, with the option for most entries of reading a more in-depth explanation. It also makes it easy to skip to different points in time, an efficiency advantage over Murphy’s online resources. Other books on the history of virology are a dense read (and lack all the fun pictures).

Murphy’s selection of images highlights his conviction that science of viral diseases predates the concept of the specificity of disease causation and depends on initial discoveries about bacteria and bacterial diseases. Readers can thus expect to see images of Pasteur, Koch, and many others who laid the groundwork for infectious disease sciences. Although in his Foreword, Murphy disclaims that his selection of names and discoveries was “completely arbitrary,” his book leaves out few major discoveries in human and veterinary virology, from Hippocrates’ observations in 400 bce to the 2010 declaration of the global eradication of rinderpest. Particularly given the origin of new, emerging, and reemerging viral infections today, it is valuable to see pictures and stories to remind us of the exceptional crossover between human and veterinary virology. Because of the author’s longstanding involvement in virology since the 1960s, his explanations for discoveries of the past 5 decades provide a unique sense of context.

This visual account of history makes obvious the sparse involvement of women in virology until the late 1900s. However, Murphy made a good effort to acknowledge women when possible. He also did a good job acknowledging seminal forces worldwide that shaped the development of virology: influential books, journals, societies, conferences, databases, even the Google search engine.

Murphy’s website (www.utmb.edu/virusimages) is surely one of the most comprehensive and publically accessible virology and history of virology resources available. But having finger-tip access to this scientist’s coffee-table book is a worthwhile investment for any infectious disease specialist interested in medical history.

